# Association of annexin A10 expression with poor prognosis of intrahepatic cholangiocarcinoma

**DOI:** 10.1186/s12885-022-09288-8

**Published:** 2022-02-28

**Authors:** Yu-Yun Shao, Hung-Yang Kuo, Yung-Ming Jeng, Yao-Ming Wu, Hsiu-Po Wang, Chiun Hsu, Chih-Hung Hsu, Hey-Chi Hsu, Ann-Lii Cheng, Zhong-Zhe Lin

**Affiliations:** 1 Graduate Institute of Oncology, National Taiwan University College of Medicine, Taipei, Taiwan; 2 Department of Oncology, National Taiwan University Hospital, Taipei, Taiwan; 3 Department of Medical Oncology, National Taiwan University Cancer Center, Taipei, Taiwan; 4 Department of Pathology and Graduate Institute of Pathology, National Taiwan University College of Medicine, Taipei, Taiwan; 5 Department of Surgery, National Taiwan University College of Medicine, Taipei, Taiwan; 6 Department of Internal Medicine, National Taiwan University College of Medicine, Taipei, Taiwan; 7 Department of Internal Medicine, National Taiwan University Hospital, Taipei, Taiwan

**Keywords:** Annexin A10, Biliary tract cancer, Cholangiocarcinoma, Prognosis, Survival

## Abstract

**Background:**

Annexin A10 expression influences the prognosis of several gastrointestinal cancers. We explored the association of annexin A10 expression with the overall survival (OS) of patients who underwent curative surgery for cholangiocarcinoma.

**Methods:**

Patients who underwent curative surgery for cholangiocarcinoma (except gallbladder cancer) and had pathological stage T1-3N0M0 disease were enrolled. Annexin A10 expression was examined by performing immunohistochemical staining. Patient demographics and survival outcome data were retrieved from medical records.

**Results:**

In total, 185 patients were enrolled. The primary tumor location was intrahepatic and extrahepatic (including the perihilar region) for 89% and 11% of patients, respectively. Positive annexin A10 staining was detected for 61 (33%) patients and associated with extrahepatic or perihilar cholangiocarcinoma (*p* = 0.001) and lower histological grade (*p* < 0.001). Patients with positive annexin A10 staining exhibited significantly poorer survival relative to patients with negative staining results (median OS, 2.5 vs. 4.9 years, *p* = 0.025). In the multivariate analysis adjusting for age, sex, tumor location, tumor grade, hepatitis infection, and disease stage, positive annexin A10 remained an independent predictor of poor OS (hazard ratio 1.572, *p* = 0.034). In the subgroup analysis, the association between annexin A10 and prognosis was restricted to intrahepatic cholangiocarcinoma. Among patients with intrahepatic cholangiocarcinoma, patients with positive annexin A10 staining exhibited significantly poorer survival compared with patients with negative annexin A10 staining (median OS, 2.3 vs. 4.9 years, *p* = 0.008).

**Conclusion:**

Positive annexin A10 expression was associated with poor prognosis of intrahepatic cholangiocarcinoma.

## Introduction

Cholangiocarcinoma arises from the biliary tract. Depending on the primary tumor location, cholangiocarcinoma can be classified as intrahepatic, perihilar, distal, or gallbladder cholangiocarcinoma. For a localized disease, curative surgical resection may be performed [1, 2]. Evan after a successful surgical resection with a clear surgical margin, recurrence of cholangiocarcinoma may occur. Several phase III clinical trials have failed to verify the benefits of adjuvant systemic therapy after resection of cholangiocarcinoma [3–6]. The identification of prognostic markers for resectable cholangiocarcinoma can aid the selection of high-risk patients for such clinical trials.

Annexins are a large family of calcium-dependent membrane-binding proteins that are involved in the cell cycle, exocytosis, and apoptosis [7, 8]. Twelve annexins have been identified in humans, and their expression varies in every organ. Among the annexin family, annexin A10 expression is well known to be the lowest on average. However, the wide application of immunohistochemical (IHC) staining with specific antibodies has demonstrated the expression of annexin A10 in normal tissues of the stomach, duodenum, urinary bladder, and kidney. It can also be expressed by oral squamous cell carcinoma, gastric carcinoma, ampullary carcinoma, pancreatic adenocarcinoma, and cholangiocarcinoma [9, 10].

The expression of short isoform mRNA of annexin A10, previously mistaken as annexin A10, was associated with favorable prognosis in hepatocellular carcinoma [11]. Expression of annexin A10 was associated with good prognosis of diffuse-type gastric carcinoma [12, 13], but poor prognosis in intestinal-type gastric carcinoma, papillary thyroid cancer, small bowel adenocarcinoma, and serous epithelial ovarian cancer [13–16]. A recent study reported that annexin A10 expression was associated with poor prognosis for the perihilar and distal cholangiocarcinoma but not for the intrahepatic cholangiocarcinoma. However, the study examined a limited tissue amount by using tissue microarray and enrolled patients with lymph node metastasis [17].

Annexin A10 can be expressed by cholangiocarcinoma, and the identification of adequate prognostic factors of cholangiocarcinoma are still required; therefore, the present study explored the association between expression of annexin A10 and survival of patients who underwent complete surgical resection for cholangiocarcinoma.

## Methods

### Patient samples

Patients who underwent potentially curative surgery for cholangiocarcinoma, except gallbladder cancer, at National Taiwan University Hospital between 1993 and 2012 were enrolled if detailed pathological data were available and regular clinical follow-ups fulfilled. Patients with stage T4 per the 6^th^ edition of the American Joint Committee on Cancer Cancer Staging Manual [18], lymph node involvement, or distant metastasis were excluded to ensure curability. Therefore, only patients with T1-3N0M0 disease were included.

All the surgical specimens were carefully re-assessed by a pathologist (YMJ) to exclude other malignancies that may arise from the biliary tracts. The specimens were anonymous and assessed without knowledge of treatment outcomes. The present study was approved by the Research Ethics Committee of National Taiwan University Hospital.

### IHC staining

IHC staining of the tissue specimens was conducted per the protocol used in other studies [9, 13]. Archival formalin-fixed paraffin-embedded tissue sections with 4-μm thickness were deparaffinized and hydrated. The tissues were then immersed in 10 mM citrate buffer (pH = 6.0) and incubated in a microwave oven at 100 °C for 10 min. Endogenous peroxidase activity was blocked by incubating the tissue slides in 0.3% hydrogen peroxide for 10 min at room temperature. The sections were then subjected to conjugation of first rabbit polyclonal anti-annexin A10 antibody (1:500; Dako Cytomation, Glostrup, Denmark) and second polyclonal goat antimouse and antirabbit immunoglobulin G antibodies (Dako Cytomation). The tissue slides were subsequently colorized with diluted 3, 3’-diaminobenzidine tetrachloride solution (Dako Cytomation) and counterstained with hematoxylin. A section of adult gastric mucosa was used as a positive control for each IHC run. Nuclear immunostaining of annexin A10 to any degree was regarded as positive [13].

### Statistical analysis

All statistical analyses were performed using SAS software version 9.4 (SAS Institute, Cary, NC, USA). A two-sided *p* value of < 0.05 was regarded as statistically significant, and that of ≥ 0.05 but < 0.10 was regarded as borderline significant. To examine the association between annexin A10 expression and patient characteristics, an independent *t* test and chi-square test were performed for continuous variables and categorical variables, respectively. The Kaplan–Meier method was used to estimate survival outcomes. To compare survival outcomes between groups, the log-rank test and a Cox proportional hazards model were used in univariate and multivariate analyses, respectively. Overall survival (OS) was defined as the period from definite tumor diagnosis until the date of death. The last follow-up date was December 31, 2020.

## Results

In total, 185 patients who received curative surgical resection for cholangiocarcinoma were enrolled in this study. The patients’ mean age was 61.7 years, and 52% of them were female (Table 1). The primary tumor locations were intrahepatic and extrahepatic (including the perihilar region) for 89% and 11% of the patients, respectively. The pathological stage was stage I, II, and IIIA for 44%, 43%, and 13% of the patients, respectively. Patients with intrahepatic cholangiocarcinoma, compared with patients with extrahepatic cholangiocarcinoma, were more likely to have hepatitis B virus infection (39% vs. 5%, *p* = 0.003).

**Table 1:** Patient characteristics and their associations with annexin A10 expression

Variables	N (%)	Annexin A10		*P*
		**Positive**	**Negative**	
**Total**	185 (100)	61 (100)	124 (100)	
**Mean age (SD, years)**	61.7 (11.4)	63.3 (11.9)	60.9 (11.2)	0.182
**Sex**				0.528
Female	97 (52)	34 (56)	63 (51)	
Male	88 (48)	27 (44)	61 (49)	
**Primary tumor location**				0.001
Intrahepatic	165 (89)	48 (79)	117 (94)	
Extrahepatic and perihilar	20 (11)	13 (21)	7 (6)	
**Tumor grade**				< 0.001
1	46 (25)	28 (46)	18 (15)	
2	46 (25)	20 (33)	45 (36)	
3	59 (32)	13 (21)	46 (37)	
4	15 (8)	0 (0)	15 (12)	
**Hepatitis virus**				
HBsAg positive	65 (35)	14 (12)	51 (41)	0.015
Anti-HCV positive	23 (12)	5 (8)	18 (15)	0.221
**AJCC stage**				0.316
I	82 (44)	24 (39)	58 (47)	
II	79 (43)	26 (43)	53 (43)	
IIIA	24 (13)	11 (18)	13 (11)	

*P* values were conducted using the independent t test for continuous variables and the Chi square test for categorical variables

*Abbreviations: SD* Standard Deviation, *HBsAg* Hepatitis B virus surface Antigen, *HCV* Hepatitis C Virus, *AJCC* American Joint Committee on Cancer

**Fig. 1:**
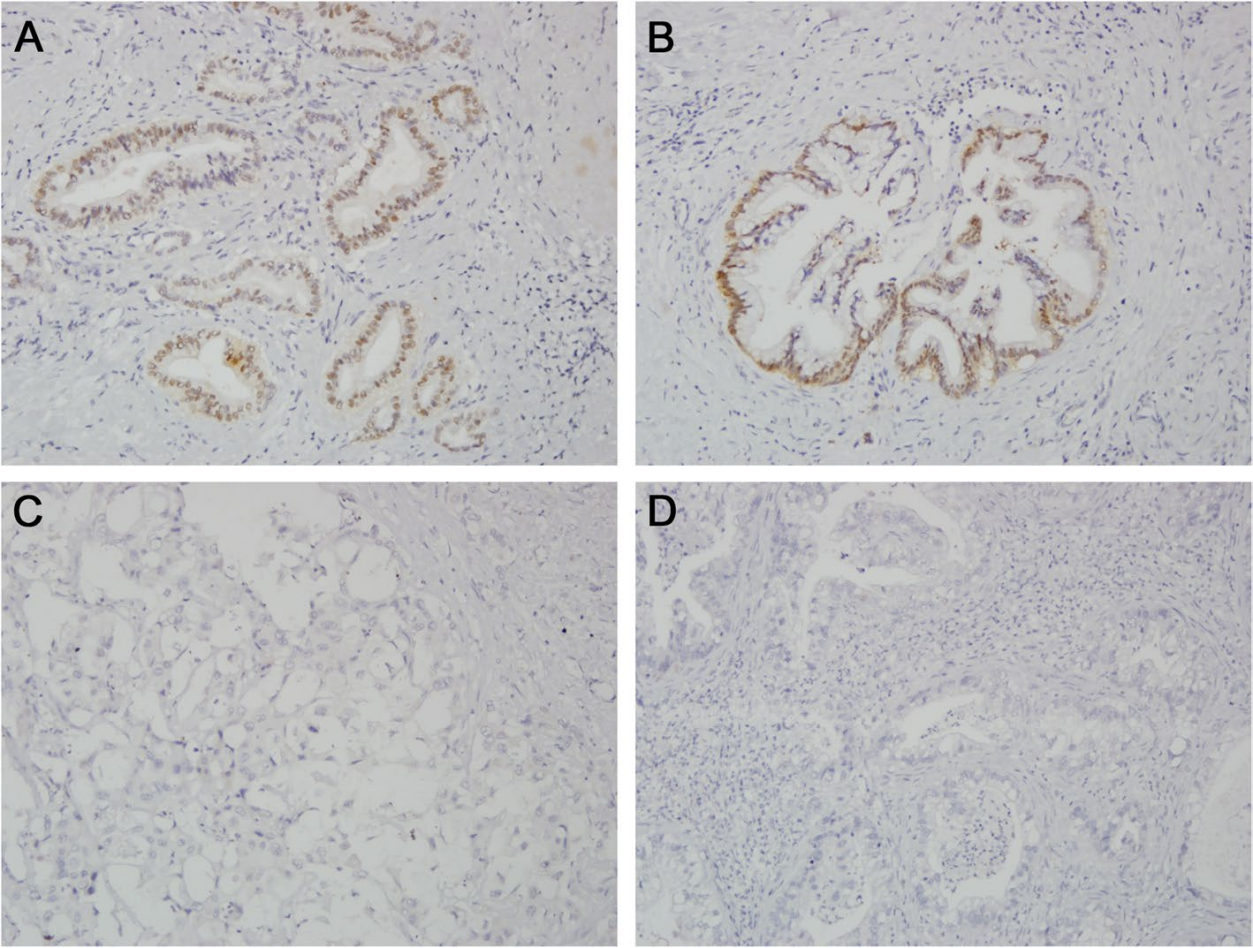
Representative photos showing **A**, **B** positive and **C**, **D** negative immunohistochemical staining of annexin A10 (200X)

The immunohistochemistry study yielded positive annexin A10 staining in the tumor tissues of 61 (33%) patients. Representative photos are shown in Fig. 1. Positive annexin A10 staining was more likely to be detected in extrahepatic or perihilar cholangiocarcinoma than intrahepatic cholangiocarcinoma (65% vs. 29%, *p* = 0.001; Table 1). Positive annexin A10 stating was also associated with lower histological grade (*p* < 0.001) and less hepatitis B virus infection (*p* = 0.015).

**Fig. 2:**
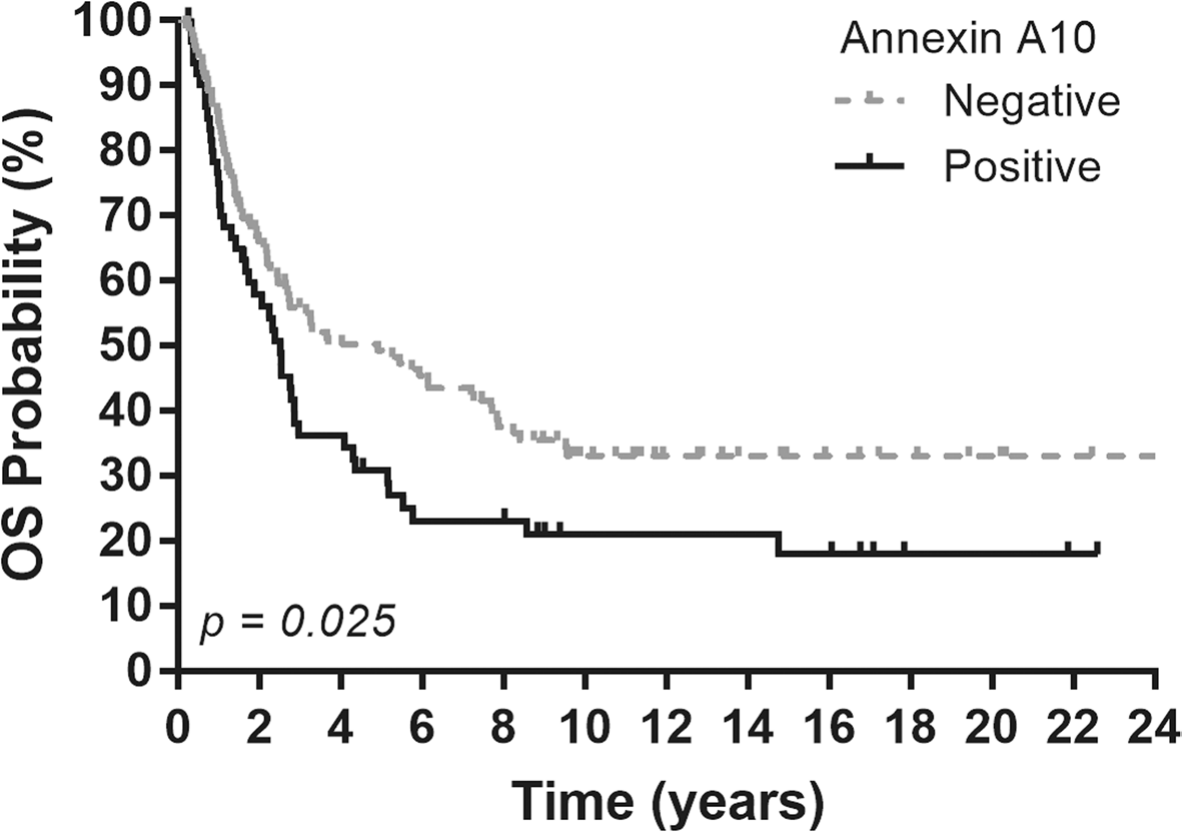
Overall survival (OS) per tumor expression of annexin A10. *P* values were conducted using the log-rank test

During the median follow-up period of 11.0 years, 120 (65%) of the patients died. The median OS was 2.9 years. Patients with positive annexin A10 staining exhibited significantly poorer survival relative to patients with negative annexin A10 staining (median OS, 2.5 vs. 4.9 years, *p* = 0.025; Fig. 2). The 5-year OS was 30.8% and 49.3% for patients with positive and negative annexin A10 results, respectively.

In the multivariate analysis adjusting for age, sex, tumor location, tumor grade, hepatitis infection, and disease stage, positive annexin A10 remained an independent predictor of poor OS (hazard ratio [HR] 1.572, *p* = 0.034; Table 2). Stage I disease was revealed to be associated with favorable OS (HR 0.482, *p* = 0.016).

**Table 2:** Univariate and multivariate analyses of potential predictors of overall survival using Cox proportional hazards models

Variables	Univariate analysis			Multivariate analysis		
	***P***	**HR**	**95% CI**	***P***	**HR**	**95% CI**
**Positive annexin A10**	0.026	1.524	1.053, 2.205	0.034	1.572	1.034, 2.390
**Male**	0.990	0.998	0.697, 1.429	0.452	1.156	0.793, 1.685
**Age**	0.174	1.012	0.995, 1.029	0.164	1.013	0.995, 1.031
**Extrahepatic and perihilar**	0.587	1.180	0.650, 2.143	0.791	0.917	0.484, 1.737
**Histological grade**	0.526	1.068	0.871, 1.310	0.1444	1.186	0.943, 1.492
**HBsAg positive**	0.456	0.866	09.594, 1.264	0.589	0.889	0.580, 1.362
**Anti-HCV positive**	0.897	0.963	0.541, 1.712	0.749	0.905	0.490, 1.669
**AJCC Stage I**	0.003	0.575	0.397, 0.831	0.016	0.482	0.267, 0.870
**AJCC Stage II**	0.071	1.392	0.972, 1.994	0.353	0.763	0.432, 1.349

*Abbreviations: HR* Hazard Ratio, *CI* Confidence Interval, *HBsAg* Hepatitis B virus surface Antigen, *HCV* Hepatitis C Virus, *AJCC* American Joint Committee on Cancer

**Fig. 3:**
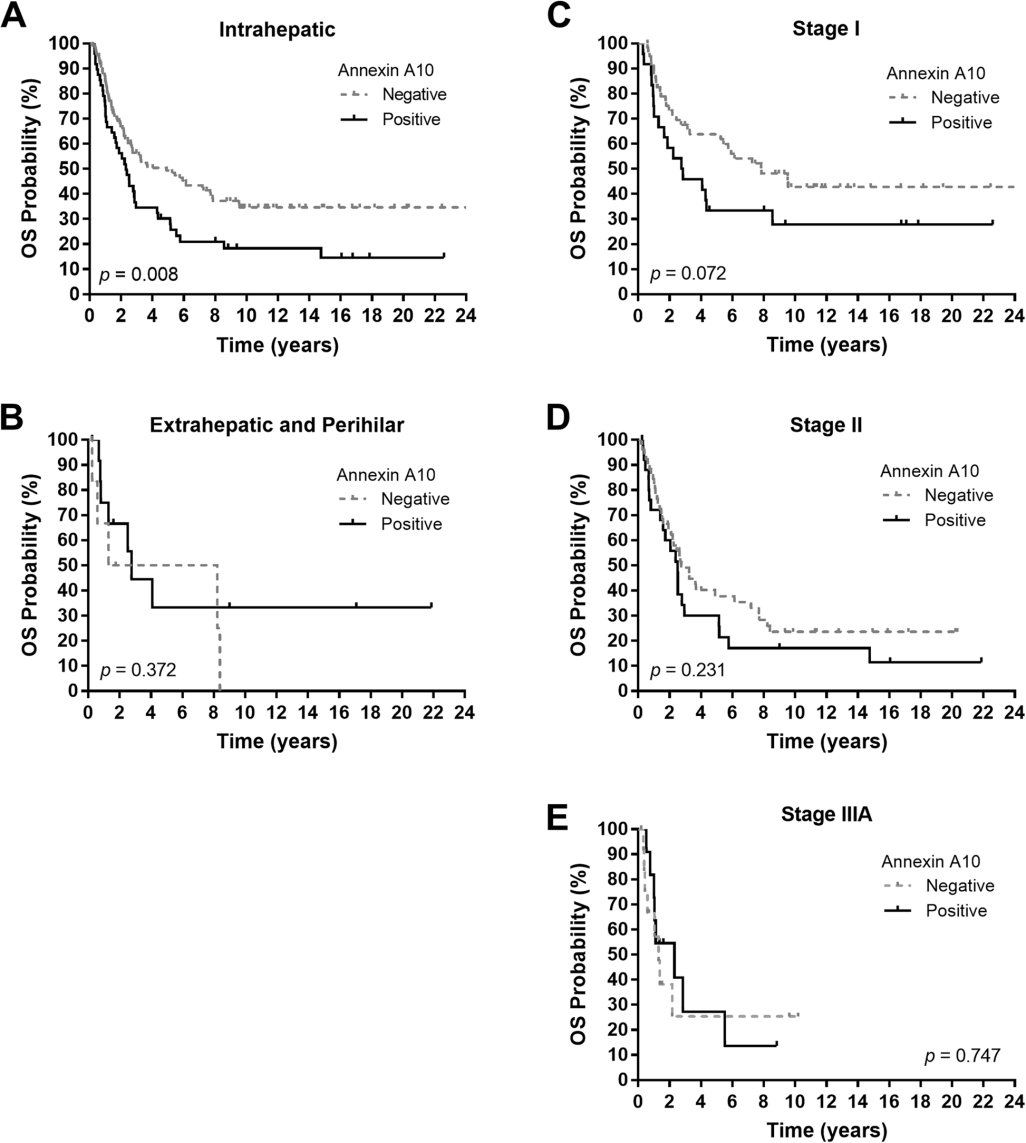
Overall survival (OS) per tumor expression of annexin A10 in patients with **A** intrahepatic tumor origin, **B** extrahepatic or perihilar origin, **C** stage I disease, **D** stage II disease, and **E** stage IIIA disease. *P* values were conducted using the log-rank test

We explored the prognosis prediction of annexin A10 in multiple subgroups. Regarding tumor location, the predictive value of annexin A10 was limited to intrahepatic tumors (Fig. 3A-B). Among patients with intrahepatic cholangiocarcinoma, patients with positive annexin A10 staining exhibited significantly poorer survival compared with patients with negative annexin A10 staining (median OS, 2.3 vs. 4.9 years, *p* = 0.008; Fig. 3A). The 5-year OS was 30.2% and 49.4% for patients with positive and negative annexin A10 results, respectively.

**Fig. 4:**
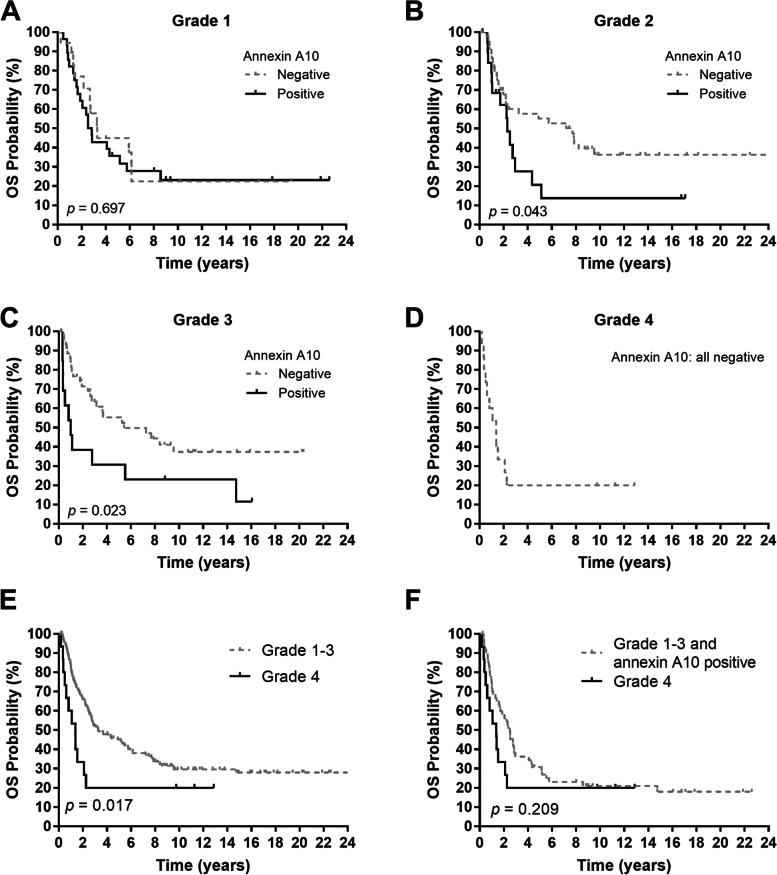
**A-D** Overall survival (OS) per tumor expression of annexin A10 in patients with cholangiocarcinoma of histological grade **A** 1, **B** 2, **C** 3, and **D** 4. **E** OS of patients according to their tumor grades (grades 1–3 vs. grade 4). **F** OS of patients with tumors of grades 1–3 and positive annexin A10 compared to patients with grade 4 tumors. *P* values were conducted using the log-rank test

Regarding disease stage, the predictive value of annexin A10 primarily resulted from stage I disease (Fig. 3D-F). The annexin A10 staining results were not associated with the prognosis of patients with histological grade 1 tumor, but a positive annexin A10 results was associated with poor prognosis for patients with grade 2 or 3 tumor (Fig. 4). All patients with grade 4 cholangiocarcinoma (*n* = 15) tested negative for annexin A10 staining; thus, the association of annexin A10 staining results with prognosis in these patients could not be analyzed. Patients with grade 4 cholangiocarcinoma exhibited significantly poorer OS than patients with grade 1–3 cholangiocarcinoma (*p* = 0.017, Fig. 4E) and similar OS compared to patients with annexin A10 positive cholangiocarcinoma (*p* = 0.209, Fig. 4F).

## Discussion

The present study revealed that tumor annexin A10 expression was associated with poor prognosis of intrahepatic cholangiocarcinoma, and this association was present in the multivariate analysis adjusting for age, sex, tumor location, tumor grade, hepatitis infection, and disease stage. Our study excluded patients with T4 disease and positive lymph node involvement, which ensured that we examined only surgeries with curative intent and a homogeneous patient group.

A previous study suggested the use of annexin A10 as a prognostic marker for cholangiocarcinoma but discovered that it had prognostic value only for perihilar and distal cholangiocarcinoma [17]. By contrast, our study indicated that positive annexin A10 predicted a poor prognosis for intrahepatic cholangiocarcinoma but not for perihilar or distal cholangiocarcinoma. The previous study included patients with lymph node involvement and distant metastasis, and it evaluated annexin A10 on the basis of both staining intensity and the percentage of positively stained cells [17]. On the contrary, we used another method to determine annexin A10 positivity, that is, nuclear staining [9]. The few patients with perihilar and distal cholangiocarcinoma in our study could also explain our inability to demonstrate the prognostic influence of annexin A10 in such patients.

In addition to having prognostic value, annexin A10 can serve as a diagnostic marker. Nuclear annexin A10 staining exhibits high specificity for adenocarcinoma of the upper gastrointestinal tract and pancreatobiliary system [9]. When staining intensity and the percentage of staining cells are considered, annexin A10 can also be used to differentiate intrahepatic cholangiocarcinoma and liver metastasis from pancreatic cancer [19].

Annexin A10 was not only associated with the prognosis of cholangiocarcinoma, but also other gastrointestinal and hepatobiliary cancers. Similar to our findings for cholangiocarcinoma, annexin A10 was associated with a poor prognosis for small bowel adenocarcinoma in another study [16]. On the contrary, annexin A10 was associated with a favorable prognosis for hepatocellular carcinoma [11, 20]. For gastric cancer, findings related to the prognostic values of annexin A10 have been inconsistent [12, 13]. Annexin A10 may have different biological functions for different malignancies; however, the various methods of assessing annexin A10 could also have contributed to the discrepancy.

Cholangiocarcinoma is a heterogeneous disease. Positive annexin A10 was most commonly detected in extrahepatic and perihilar cholangiocarcinoma; this finding was compatible with that of a previous study [9]. Although annexin A10 staining was positive in only 29% of patients with intrahepatic cholangiocarcinoma, its prognostic influence was strongest for this location relative to other primary tumor locations. Positive annexin A10 was more frequently observed in grade 1 tumor (61%) relative to grade 2 (31%) and grade 3 (22%) tumor. However, the prognostic influence of annexin A10 was stronger for grade 2 and 3 tumors.

Our study had a few limitations. We lacked a validation cohort; hence, our findings are, at best, hypothesis generating. However, we avoided multiple comparison bias because our assessment of annexin A10 positivity was clear and followed the protocol used in a previous study [9]. Although the total number of patients examined in our study was high, the percentage of patients with extrahepatic and perihilar cholangiocarcinoma was low. This could have contributed to the lack of prognostic power of annexin A10 for these tumors. Similarly, only 15 patients with grade 4 tumors were included, so the association of annexin A10 expression with prognosis could not be analyzed in these patients. However, the low patient number with grade 4 tumors reflected the aggressive nature of such undifferentiated tumors and the low possibility of curative surgery upon diagnosis.

## Conclusions

Positive annexin A10 expression was associated with poor prognosis of intrahepatic cholangiocarcinoma.

## Data Availability

All data generated or analyzed during this study are included in this published article.

## Abbreviations

IHC: Immunohistochemical
OS: Overall survival
HR: Hazard ratio
